# Pattern of dental needs and advice on Twitter during the COVID-19 pandemic in Saudi Arabia

**DOI:** 10.1186/s12903-021-01825-4

**Published:** 2021-09-17

**Authors:** Khalifa S. Al-Khalifa, Eman Bakhurji, Hassan S. Halawany, Esraa M. Alabdurubalnabi, Wejdan W. Nasser, Ashwin C. Shetty, Shazia Sadaf

**Affiliations:** 1 Department of Preventive Dental Sciences, College of Dentistry, Imam Abdulrahman Bin Faisal University, Dammam, Saudi Arabia; 2 Department of Periodontics and Community Dentistry, College of Dentistry, King Saud University, Riyadh, Saudi Arabia; 3 Dental Internship Program, College of Dentistry, Imam Abdulrahman Bin Faisal University, Dammam, Saudi Arabia; 4 Department of Dental Education, College of Dentistry, Imam Abdulrahman Bin Faisal University, Dammam, Saudi Arabia

**Keywords:** COVID-19, Twitter, Pandemic, Oral health, Dentistry, Social media, Tweet, Saudi Arabia

## Abstract

**Aim:**

To compare and evaluate the influence of the COVID-19 outbreak on tweets related to dental treatment needs and advice of Saudi Twitter users in 2020 by comparing them to the same time-period in 2019.

**Methods:**

Eight independent searches based on dentistry related keywords: “teeth, mouth and gingiva” were carried out within the timeframe between the 23rd of March and the 21st of June for the years 2020 and 2019. Extracted tweets were analyzed by two calibrated examiners as tweets containing expressed dental needs and tweets for dental advice, while spam tweets were excluded. Descriptive analysis was performed to present the overview of the findings using SPSS. Bivariate analysis was performed with Pearson’s Chi Square, Fisher’s Exact test and Mann–Whitney U test. Statistical significance was set at p ≤ 0.05.

**Results:**

A total of 595 tweets from the year 2019 and 714 tweets from the year 2020 were obtained. Overall, combined dental needs and advice tweets, retweets, likes, and replies were higher in 2020 compared to 2019. Dental needs tweets were higher in 2020 compared to 2019, while dental advice tweets were lower in 2020 compared to 2019. Statistically significant differences were found between 2020 and 2019 with regards to dental needs well as with dental advice (p < 0.05). In addition, statistically significant differences were found between 2019 and 2020 with presence of pain, urgency of the dental need and type of advisor (p < 0.05).

**Conclusion:**

An obvious impact of the pandemic can be seen in the form of increased self-reported dental needs, pain and urgency among the public in Saudi Arabia. This study highlights the importance of social media, specifically Twitter, in expressing the public needs and utilizing it as a platform for education and advice.

## Introduction

The COVID-19 pandemic has swept the entire world across 223 countries resulting in 163,869,893 confirmed cases of infection and 3,398,302 deaths worldwide as of the 20th of May, 2021 [1]. Governments around the world have imposed strict measures to ensure social distancing and preventive protocols while effective therapeutics and vaccines are being deployed to control the pandemic and bring life back to normal.

Apart from infecting populations and increasing the disease burden, COVID-19 has posed a significant threat to the healthcare professionals in the field. Among the most affected are the frontline workers and dentists with a greater risk of transmission of SARS-CoV-2 through the saliva of patients who are infected and the transmission of COVID-19 through asymptomatic carriers or potential patients in incubation status. Studies have reported increasing concern amongst dentists of acquiring COVID-19 during dental procedures. This had led to suspension of dental practices during the outbreak of the pandemic [2, 3].

In Saudi Arabia, dental practices were suspended from the middle of March till August 2020 with strict preventive measures across the country for only emergency procedures. This has forced patients requiring routine dental services to seek consultations through social media platforms and benefit from teledentistry [4, 5].

Information acquired through the internet and the increased use of social media by patients seeking consultations have been reported during outbreaks in recent times [2]. This was witnessed again during the lockdowns due to the COVID-19 where significant surge in use of social media was observed [6]. Studies have reported analysis of social media usage by healthcare providers for drug adverse events detection, assessment of public opinion about health-related issues such as vaccination and infectious disease outbreak surveillance and in developing public health policies [2, 6].

In Saudi Arabia currently 25 million (72.38%) of the population are active social media users with 20.3 million (58%) of Saudis on Twitter [7]. Twitter is a free social networking website enabling users to post “Tweets” which is a short text message limited to 140 characters using the web, instant online message, or mobile phone. Statistics show more than 500 million people are active on Twitter generating more than 340 million tweets and 1.6 billion search queries per day. Healthcare professionals across the globe have been found to use Twitter to reach out to their colleagues, patient population and others collecting real-time health data and gaining more insight on trends in healthcare. Several studies have examined the content of health-related tweets on Twitter [5, 8, 9].

This study aimed to compare and evaluate the influence of the COVID-19 outbreak on the tweets related to the dental treatment needs and advice of Saudi Twitter users in 2020 by comparing them to the same time-period in 2019.

## Materials and methods

This infoveillance study was developed to assess the activity of Twitter users in Saudi Arabia regarding dental treatment needs and advice during the COVID-19 pandemic in 2020 comparing it with the activity in the same period in 2019. This study was performed using similar methods to those used in previous studies which assessed the use of Twitter during the COVID-19 pandemic [2, 9, 10]. Since this research used publicly available data that did not involve human subjects; thus, it did not require an institutional review board approval from Imam Abdulrahman Bin Faisal University, Dammam, Saudi Arabia.

### Search strategy

Eight independent searches based on dentistry related keywords: [“أسنان” teeth, “اسنان” teeth,” سنون” teeth, “ضروس” molars, “ضرس” molar, “لثة” gingiva, “اللثة” gingiva, “فم” mouth] were carried out on the 22nd of December 2020. The tweets were extracted within the timeframe between the 23rd of March and the 21st of June for both 2020 (exposed to Coronavirus “COVID-19″) and 2019 (unexposed). This timeframe reflects the period which the Saudi Arabian government implemented a complete lockdown on the country. The Twitter data collected included: the tweet’s ID, full text content, username, user location, and the count of retweets, quotes, replies and likes. It was collected using a Python-based library snscrape [11], a scraper for social networking services (SNS) that permits the retrieval of older tweets.

Using each key term individually resulted in 121 K tweets from the year 2019, 171 K tweets from the following year of 2020 with duplicates removal. However, since the Arabic language is used across many countries, the large amount of data did not represent the collected views from the locals of Saudi Arabia. Therefore, the data were filtered to include tweets that have any of the following terms in their location ["سعودي", " SA”, "KSA"]. As a result, the amount of data decreased to 8K and 12K tweets from 2019 and 2020, respectively.

In addition, to prevent opinion bias occurring from the repetitive tweets from the same user, the data were filtered to limit data to include only the first occurring tweet per username. This resulted in 4 K and 5 K for years 2019 and 2020, respectively. Also, data were filtered based on the content, where only unique tweet content was included. Furthermore, content was cleaned of numbers and non-Arabic characters.

### Coding of tweets

Extracted tweets were categorized into either: (1) tweets containing expressed dental needs (2) tweets on dental advice, while spam tweets of irrelevant content to the scope of the study were excluded. Included tweets for dental needs were coded according to the presence or absence of pain, expressed urgency and the type of dental need (esthetics, dental, surgical or nonspecific). On the other hand, advice tweets were coded according to type of dental advice given (esthetics, dental, surgical or nonspecific) as seen in Table 1. Furthermore, dental advice was categorized based on the type of provider either dental or non-dental professional.

**Table 1:** Classification of tweets related to dental needs and dental advice

Category		Example
*Dental needs*		
Presence/absence of pain		“I have a dental appointment and I’m afraid to go, who dares and goes out in this pandemic? If it wasn’t urgent, I wouldn’t have gone but the pain is killing me”
Expressed urgency		“Do you have dental ER clinic? Please answer”
Types of dental needs	Esthetics	“Kindly answer me with honesty, who knows a dentist that is good and has integrity especially for anterior teeth build ups?”
	Dental	“I want the best dental clinic in Riyadh. I have two teeth for restoring. And do they deal with instalment payments if it was expensive?”
	Surgical	“Can you ask them? I want a good dentist that does not hurt while working on wisdom teeth and restorations”
	Undefined	“If I may ask? we want a dental clinic that is open during lockdown hours in Riyadh “
*Dental advice*		
Types of dental advice	Esthetics	“First of all, you need dental prophy, if you have spaces, you can start braces treatment. If your teeth are aligned then proceed with teeth whitening”
	Dental	“If it doesn’t improve in a week, the problem could need a root canal and that’s when you must visit a dentist. Make sure to choose a clinic that takes precautionary measures seriously”
	Surgical	“It’s okay, you don’t have to treat the problem. Those are primary teeth will be extracted and replaced”
	Undefined	“I’m a dentist and I agree although most doctors don’t like natural remedies. But some help with healing ulcers”

The coding was performed by two trained and calibrated investigators (EA and WN) with experience in dentistry. Both coders conducted a pilot by coding 100 random posts. A weighted Kappa was utilized to test the reliability. Both investigators classified another 200 randomly selected tweets and reclassified one week after the first coding. The weighted Kappa was 0.927 for inter-agreement and 0.912 (EA) and 0.843 (WN) for intra-agreement with an overall excellent reliability. Any conflict in coding was resolved by a gold standard examiner (KK) via discussion to ensure full understanding of tweets categories and themes by both investigators.

### Analysis

Statistical analysis for this study was carried out using SPSS version 20.0 (SPSS Inc., Chicago, II, USA). Descriptive analysis was performed to present the overview of the findings. The results were presented in the form of frequency tables with percentages and mean (SD) as well as graphical presentation in the form of line charts. Bivariate analysis was performed with Pearson’s Chi Square, Fisher’s Exact test and Mann–Whitney U test. Statistical significance was set at p ≤ 0.05.

## Results

**Fig. 1:**
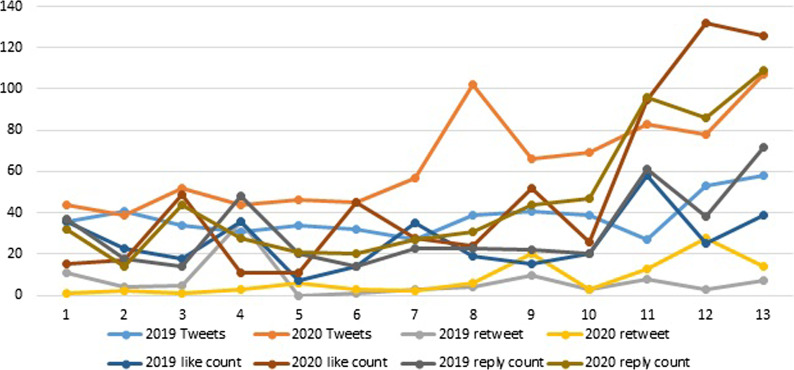
Trends in combined dental needs and advice tweets, retweets, likes, and replies from March 23 to June 21, 2019 and 2020 (COVID-19 outbreak)

A total of 595 tweets were obtained between March 23 and June 21, 2019 and 714 were retrieved from the same days of 2020, totaling 1,309 were selected. Most of the tweets were from the Western and Riyadh regions. Figure 1 displays trends in dental needs and advice tweets, retweets, likes, and replies from March 23 to June 21, 2019 and 2020 (COVID-19 outbreak). Overall, combined dental needs and advice tweets, retweets, likes, and replies were higher in 2020 compared to 2019.

**Fig. 2:**
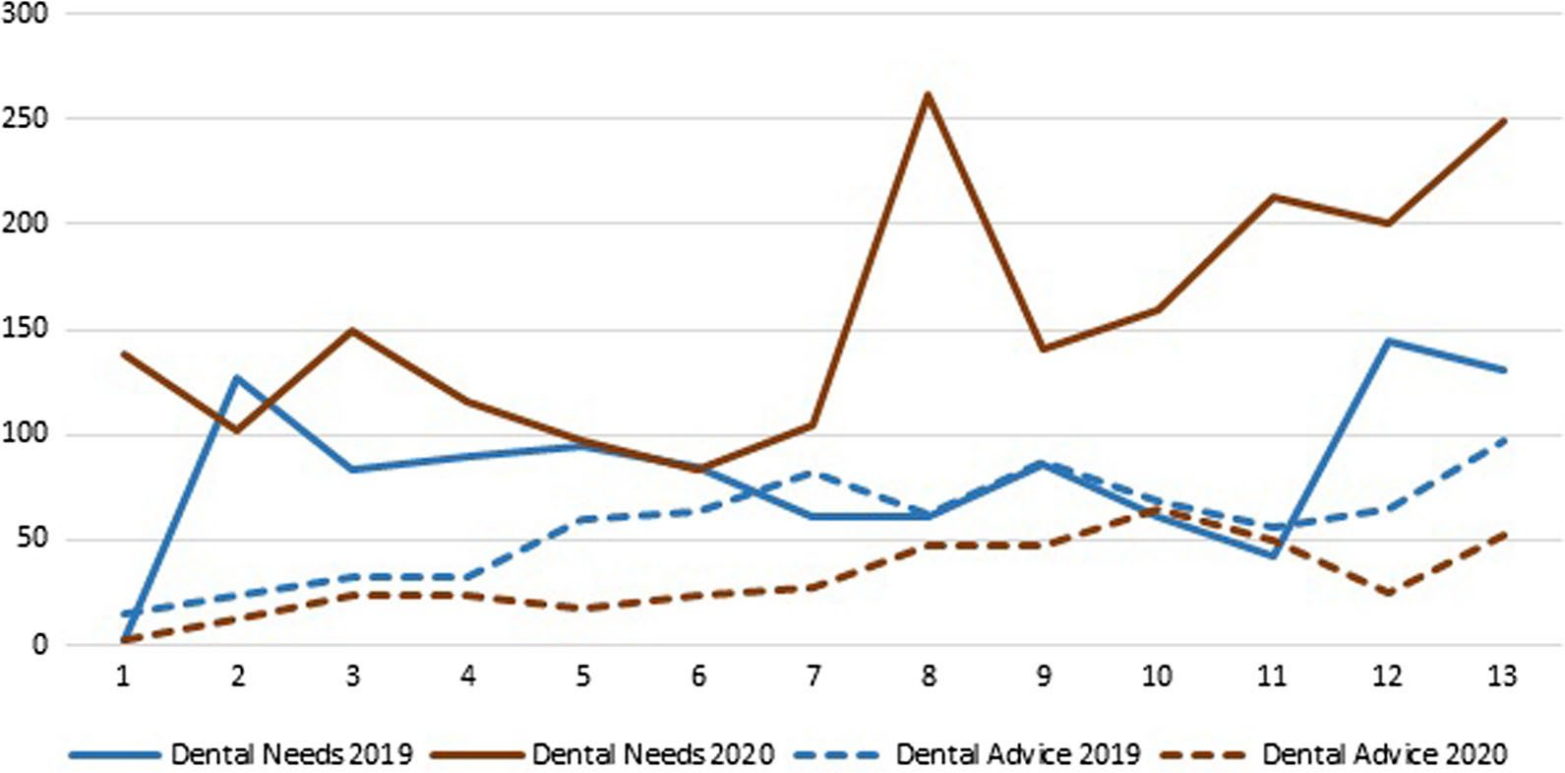
Distribution of tweets related to dental needs and dental advice from March 23 to June 21, 2019 and 2020 (COVID-19 outbreak)

When tweets were divided between dental needs and advice, dental needs tweets were higher in 2020 compared to 2019, while dental advice tweets were lower in 2020 compared to 2019 (Fig. 2).

Table 2 shows dental needs and advice as they are further distributed according to the predefined classifications. Among the defined dental needs and types of advice, most were surgical in both 2019 and 2020. Statistically significant differences were found between dental needs and year as well as with dental advice and year (p < 0.05).

**Table 2:** Distribution of the tweets according to the type of dental treatment needed for both dental needs and advice in 2019 and 2020

		n (%)		p value
		2019	2020	
Dental needs	Esthetics	47 (12.8)	46 (7.6)	0.004*
	Dental	54 (14.7)	79 (13.1)	
	Surgical	74 (20.1)	96 (15.9)	
	Undefined	193 (52.4)	381 (63.3)	
	Total	368 (100)	602(100)	
Dental advice	Esthetics	14 (6.2)	14 (12.5)	0.014*
	Dental	22 (9.7)	12 (10.7)	
	Surgical	77 (33.9)	21 (18.8)	
	Undefined	114 (50.2)	65 (58.0)	
	Total	227 (100)	112 (100)	

*Statistically significant at p ≤ 0.05

Table 3 display differences between years 2019 and 2020 according to the predefined classifications; presence of pain, urgency of the dental need, dental advice and type of advisor. Statistically significant differences were found between 2019 and 2020 with presence of pain, urgency of the dental need and type of advisor (p < 0.05).

**Table 3:** Differences in the dental tweets between years 2019 and 2020 according to the predefined classifications

		n (%)		p value
		2019	2020	
Presence/absence of pain	Yes	86 (23.4)	213 (35.4)	< 0.001*
	No	282 (76.6)	389 (64.6)	
Expressed urgency	Yes	25 (6.8)	96 (15.9)	< 0.001*
	No	343 (93.2)	506 (84.1)	
Advice	Yes	227 (27.3)	112 (22.8)	0.078
	No	605 (72.7)	380 (77.2)	
Advisor	Dental	0 (0.0)	12 (5.3)	0.010*
	Non-dental	110 (100.0)	215 (94.7)	

*Statistically significant at p ≤ 0.05

Furthermore, statistically significant difference in the mean (SD) retweet and reply count was found between dental and non-dental advisors in 2020 (p < 0.05). The mean (SD) retweet and reply count in 2020 was higher among dental advisors as seen in Table 4.

**Table 4:** Comparison of dental advice retweets, likes, and replies from March 23 to June 21, 2019 and 2020 (COVID-19 outbreak) by type of advisor

		2019		2020	
		Mean (SD)	p value	Mean (SD)	p value
Retweet	Dental	0.5 (1.0)	0.373	0.3 (0.5)	0.002*
	Non-dental	0.2 (0.7)		0.1 (1.2)	
Like	Dental	1.3 (2.5)	0.918	0.9 (1.7)	0.086
	Non-dental	1.0 (3.9)		0.8 (3.1)	
Reply	Dental	1.5 (2.4)	0.647	0.7 (0.7)	0.004*
	Non-dental	1.2 (3.5)		0.3 (0.5)	

*Statistically significant at p ≤ 0.05

## Discussion

The pandemic outbreak of COVID-19 has affected all people globally. It has changed the way people communicate, interact, and express their thoughts and needs. This infoveillance study assessed self-reported dental needs posted on the social media platform, Twitter, in 2020 during COVID-19 outbreak and compared it to the same time in 2019 in Saudi Arabia. The study found that tweets, retweets, and replies on dental needs have increased drastically during the pandemic period. Additionally, retweets during the pandemic were more likely to be from dental advisors’ accounts than non-dental advisors in comparison to the same period in 2019. This study highlights the importance of social media platforms in assessing needs and demands of the public. It also highlights the impact that these platforms could have on its viewers which can be utilized in providing education and advice on oral health.

The number of tweets on self-reported dental needs, specifically on dental pain, and urgent dental issues have dramatically increased during the pandemic period. In contrast, tweets on self-reported esthetic dental needs have decreased in comparison to the same time in 2019. These findings are similar to a Brazilian infoveillance study that assessed self-reported dental needs reported on Twitter during the pandemic [10]. The authors of the study attributed the increased number of tweets on dental pain and urgent dental needs to the psychological impact of the pandemic which have shifted the public to social media for communication. This explanation is true especially  as social distancing has changed the dynamics of people’s communication channels in a way that forced them unconsciously to be more engaged and active on social media.

Facebook, WhatsApp, Instagram and Twitter became more powerful during the pandemic for individuals to access real health information related to COVID -19 rather than following fake news [12].

The COVID-19 pandemic crisis could have created a tremendous amount of anxiety, distress and isolation that can be expressed virtually through personal accounts on social media. A recent study reported moderate to severe psychological impact of COVID -19 in the Saudi population [13]. A further study also reported depression and high prevalence of distress in the Saudi population during outbreak [14]. This emerging tendency for anxiety and depression which resulted in increased use of social media during the pandemic was also experienced globally. A number of studies reported increased online use and access to social media during the outbreak whether in China where the pandemic originated or from other parts in the world [2, 9, 15].

Besides the psychological impact of the pandemic that resulted in increased social media use, businesses and social activities were shut down in Saudi Arabia during the lockdown period which have created even more time for young adults to be connected on their social media accounts. The lockdown that lasted for few months in Saudi Arabia starting from March 2020 could also explain the trends we see in Twitter with regards to dental needs including pain and urgency. It could also explain the significant increase in retweets of dental advice, likes and replies from dental advisors’ accounts. Dental clinics are considered one of the most high-risk practices for cross-infection due to the production of aerosols and the close contact to the patient’s oral cavity and respiratory system [16, 17]. Therefore, dental clinics were among the businesses that were limited to emergency procedures during the outbreak in Saudi Arabia [18]. That could have made the general public shy away from visiting the dentists even for urgent dental pain as they were afraid of the spread of infection since COVID-19 is more infectious in the dental environment. It could also explain the significant increase in dental retweets from dental advisors during the outbreak because patients were turning to dental accounts to get advice. On the other hand, dentists were more available on their social media to provide advice since most dental clinics were not-operatable during the outbreak. Furthermore, dental advices were paid more attention during the outbreak as they originated from dental professional Twitter accounts.

One lesson learnt from social distancing during the pandemic is adaptation. The shift in social communication from physical to virtual during the outbreak could be viewed as the highlight of the pandemic. Social media became a venue for many young adults to vent their feelings and to communicate. Therefore, social media becomes very important in delivering health messages to the public while the role of dentists in disseminating credible information to the general population through social media becomes vital. It has been used in delivering and obtaining health information among different populations such as special needs, HIV and diabetic patients [19, 20], and recently social media has been utilized to obtain information regarding COVID-19 as well [12, 21]. The public, on the other hand, were found willing to obtain oral health information through social media, especially young individuals [22]. Therefore, the pattern seen in this study of online activity and social media use to deliver and obtain oral health advice should continue even after the pandemic to educate the public through social media. However, it should be practiced with caution since social media platforms are not well controlled or monitored and are full of biased and false information [23].

Another sign of adaptation to the pandemic is the emergence of what is called “teledentistry”. Teledentistry is a combination of tele-communication and dentistry. It involves remote consultation through exchange of information and images between the patient and the dentist to provide treatment plan or at least management of dental problems if possible [24]. Traditionally, teledentistry is used to assist underserved patients and those who live in rural areas or remote places [25]. This concept becomes handy during the pandemic where many can get dental consultations and limited dental services remotely. Although dental professionals in Saudi Arabia believed that teledentistry would improve dental care, only 50% have applied it in their practice [4, 26]. Despite that dentists are being skeptical about the application of teledentistry in dental care, teledentistry provides a safe venue for patients to get consultations and limited dental care during the pandemic in a safe and convenient manner.

A limitation of this study is relying on self-reported dental needs posted on Twitter which might not reflect actual needs. However, it still could be an indicator of perceived dental needs. Additionally, retweeting and liking dental advices on the social network does not guarantee that these advices are being followed. However, consistent messages over time and even post-pandemic could at least spread awareness regarding oral health among the public.

Future studies are needed to investigate the implied sentiments in the tweets to further analyze the psychological aspects of those reporting dental treatment needs. The dental advice can be further assessed by the type of advisor to explain the rationale for provision of dental advice and the background of the dental advisor whether professional or not. Furthermore, dental needs and advice after the lockdown can be compared to the timeframe examined in this study.

## Conclusion

This study highlights the importance of social media, specifically Twitter, in expressing the public needs and utilizing it as a platform for education and advice. It also highlights the impact of the pandemic that can be seen in the increased self-reported dental needs, pain and urgency among the public in Saudi Arabia. An increased number of retweets, likes and replies on tweets of dental advices from dental advisors in 2020 in comparison to 2019 indicates awareness of the public in obtaining dental information from the right recourses and trusting credible evidence from dental accounts.

## Data Availability

The data supporting the findings of this study are available upon reasonable request from the corresponding author.
