# Correction to: Pattern of dental needs and advice on Twitter during the COVID-19 pandemic in Saudi Arabia

**DOI:** 10.1186/s12903-021-01842-3

**Published:** 2021-09-29

**Authors:** Khalifa S. Al-Khalifa, Eman Bakhurji, Hassan S. Halawany, Esraa M. Alabdurubalnabi, Wejdan W. Nasser, Ashwin C. Shetty, Shazia Sadaf

**Affiliations:** 1 Department of Preventive Dental Sciences, College of Dentistry, Imam Abdulrahman Bin Faisal University, Dammam, Saudi Arabia; 2 Department of Periodontics and Community Dentistry, College of Dentistry, King Saud University, Riyadh, Saudi Arabia; 3 Dental Internship Program, College of Dentistry, Imam Abdulrahman Bin Faisal University, Dammam, Saudi Arabia; 4 Department of Dental Education, College of Dentistry, Imam Abdulrahman Bin Faisal University, Dammam, Saudi Arabia

## Correction to: BMC Oral Health (2021) 21:456 https://doi.org/10.1186/s12903-021-01825-4

After publication of the original article [1], an error was identified in the first paragraph of ‘Search Strategy’ section:

The paragraph currently reads:

Eight independent searches based on dentistry related keywords: [“نانسأ “teeth, “نانسا “teeth,” نونس “teeth, “ةثللا”,gingiva” ةثل”,molar” سرض”,molars” سورض” gingiva, “مف “mouth] were carried out on the 22nd of December 2020. […].

Terefore, the data were fltered to include tweets that have any of the following terms in their location ["يدوعس ", "SA”, "KSA"]. As a result, the amount of data decreased to 8 K and 12 K tweets from 2019 and 2020, respectively.

The paragraph should read:

Eight independent searches based on dentistry related keywords: [“أسنان” teeth, “اسنان” teeth,” سنون” teeth, “ضروس” molars, “ضرس” molar, “لثة” gingiva, “اللثة” gingiva, “فم” mouth] were carried out on the 22nd of December 2020. […].

Therefore, the data were filtered to include tweets that have any of the following terms in their location ["سعودي", "SA”, "KSA"]. As a result, the amount of data decreased to 8 K and 12 K tweets from 2019 and 2020, respectively.

The original article has been corrected.
